# Active Ingredients and Mechanisms of Change in Motivational Interviewing for Smoking Cessation in Patients With Coronary Artery Disease: A Mixed Methods Study

**DOI:** 10.3389/fpsyg.2021.599203

**Published:** 2021-06-22

**Authors:** Jos Dobber, Marjolein Snaterse, Corine Latour, Ron Peters, Gerben ter Riet, Wilma Scholte op Reimer, Lieuwe de Haan, Berno van Meijel

**Affiliations:** ^1^Faculty of Health, Center of Expertise Urban Vitality, Amsterdam University of Applied Sciences, Amsterdam, Netherlands; ^2^Amsterdam University Medical Centers, Department of Cardiology, University of Amsterdam, Amsterdam, Netherlands; ^3^Amsterdam University Medical Centers, Department of Psychiatry, University of Amsterdam, Amsterdam, Netherlands; ^4^Inholland University of Applied Sciences, Amsterdam, Netherlands; ^5^Amsterdam University Medical Centers, Department of Psychiatry, VU Medical Center, Public Health Research Institute (APH), Amsterdam, Netherlands; ^6^Parnassia Psychiatric Institute, The Hague, Netherlands

**Keywords:** coronary artery disease, smoking cessation, motivational interviewing, mechanism of change, active ingredients

## Abstract

**Background:** For patients with coronary artery disease (CAD), smoking is an important risk factor for the recurrence of a cardiovascular event. Motivational interviewing (MI) may increase the motivation of the smokers to stop smoking. Data on MI for smoking cessation in patients with CAD are limited, and the active ingredients and working mechanisms of MI in smoking cessation are largely unknown. Therefore, this study was designed to explore active ingredients and working mechanisms of MI for smoking cessation in smokers with CAD, shortly after a cardiovascular event.

**Methods:** We conducted a qualitative multiple case study of 24 patients with CAD who participated in a randomized trial on lifestyle change. One hundred and nine audio-recorded MI sessions were coded with a combination of the sequential code for observing process exchanges (SCOPE) and the motivational interviewing skill code (MISC). The analysis of the cases consisted of three phases: single case analysis, cross-case analysis, and cross-case synthesis. In a quantitative sequential analysis, we calculated the transition probabilities between the use of MI techniques by the coaches and the subsequent patient statements concerning smoking cessation.

**Results:** In 12 cases, we observed ingredients that appeared to activate the mechanisms of change. Active ingredients were compositions of behaviors of the coaches (e.g., supporting self-efficacy and supporting autonomy) and patient reactions (e.g., in-depth self-exploration and change talk), interacting over large parts of an MI session. The composition of active ingredients differed among cases, as the patient process and the MI-coaching strategy differed. Particularly, change talk and self-efficacy appeared to stimulate the mechanisms of change “arguing oneself into change” and “increasing self-efficacy/confidence.”

**Conclusion:** Harnessing active ingredients that target the mechanisms of change “increasing self-efficacy” and “arguing oneself into change” is a good MI strategy for smoking cessation, because it addresses the ambivalence of a patient toward his/her ability to quit, while, after the actual cessation, maintaining the feeling of urgency to persist in not smoking in the patient.

## Introduction

About 40% of smokers successfully quit smoking immediately after experiencing an acute coronary syndrome (ACS) (Snaterse et al., 2015). Still, about 25% (Snaterse et al., 2018) −43% (Snaterse et al., 2015; Kotseva et al., 2019) do not undertake any attempt to quit and persist in smoking. These are noteworthy statistics since smoking cessation strongly reduces the risk of a recurrent myocardial infarction in this high-risk population (OR = 0.57; 95% confidence interval (CI) 0.36–0.89) (Chow et al., 2010).

Motivational interviewing (MI) (Miller and Rollnick, 2013) may enhance the willingness and the ability of the persistently smoking patients to quit. Miller and Rollnick (Miller and Rollnick, 2013, p. 29) define MI as “a collaborative, goal-oriented style of communication with particular attention to the language of change. It is designed to strengthen personal motivation for and commitment to a specific goal by eliciting and exploring the person's own reasons for change within an atmosphere of acceptance and compassion.” MI is a psychological intervention to enhance the intrinsic motivation for behavior change, and it addresses the ambivalence, willingness, ability, and readiness of the patient to change. Many patients are ambivalent about smoking cessation: e.g., they may be willing to quit smoking for their health, but at the same time, they consider smoking as a stress reducing activity; or they are willing are but not (yet) able to stop smoking. The impact of MI on smoking cessation in patients shortly after a cardiovascular event is thought to be due to its focus on the willingness of the patient to quit smoking, and on the ambivalence, ability, and readiness of the patient. In MI, a health professional evokes and strengthens the motives for change (“change talk”) of the patient and tries to reduce or to soften the “sustain talk” of the patient, i.e., the statements of the patient in favor of status quo. Thus, the MI practitioner intentionally influences the willingness, ability, and readiness to change. The MI-communication style is empathetic, and in accordance with “MI Spirit,” which refers to the MI-core values of partnership, acceptance, evocation, and compassion (Miller and Rollnick, 2013). Four overlapping central processes support the MI practitioner to navigate the MI sessions: engaging (establishing a trusting relationship), focusing (concentrating on the change goal), evoking (helping the patient to find and to voice his/her own motives for change), and planning (developing a commitment to change and creating a specific action plan) (Miller and Rollnick, 2013).

In a meta-analysis of 28 studies, Lindson Hawley et al. (2015) found a significant effect on smoking cessation using MI compared to a piece of brief advice or usual care (RR = 1.26; 95% CI 1.16–1.36), and, in another meta-analysis of eight studies, Lundahl et al. (2013) also found a significant effect in favor of MI compared to unspecified other smoking cessation interventions in medical care settings (OR = 1.34; 95% CI 1.05–1.70). Four of the studies included in the latter meta-analysis were also included in the meta-analysis by Lindson Hawley et al. (2015). However, there was considerable clinical heterogeneity (patient populations, settings, intervention content, and control interventions) and heterogeneity of effect sizes in both meta-analyses, and only one study pertained to patients with coronary artery disease (CAD). We found three randomized clinical trials (RCTs) estimating the effects of MI on smoking cessation in patients with CAD (Table 1); two of these studies have not been included in the meta-analyses. Dornelas et al. (2000) reported a difference in the cessation rate, 55% for the MI group vs. 34% in the minimal care group after 1 year. This study was also included in the meta-analysis by Lindson Hawley et al. (2015). Bredie et al. (2011) found a statistically significant difference in smoking cessation in favor of nurse-based motivational interviewing (NBMI 26% quitters; care as usual 7%). In an RCT on the effect of a minimal intervention strategy for cardiology patients (C-MIS), which includes elements of motivational interviewing, Wiggers et al. (2006) found no significant difference in smoking cessation [abstinence rate nicotine replacement therapy (NRT) and C-MIS 28%, NRT only 24%; absolute risk reduction (ARR) = 0.04; 95% CI −0.06 to 0.14]. So, MI may be effective for smoking cessation in patients with CAD, but the research on MI in this patient group is scarce and inconclusive.

**Table 1:** Overview of randomized trials of MI on smoking cessation in patients with CAD.

**Study**	**Quitters/*n* experimental group**	**Quitters/*n* control group**	**Risk difference (95% confidence interval)**	**Time to follow up (months)**	**Method of smoking status verification**
Dornelas et al. (2000)	28/54	16/46	0.17 (−0.2; 0.36)^a^	12	Self-report
Wiggers et al. (2006)	38/137	32/132	0.04 (−0.06; 0.14)	12	Biochemical marker
Bredie et al. (2011)	12/46	3/42	0.19 (0.04; 0.34)^a^	3	Self-report

a *For this study, we calculated the risk difference and the 95% confidence interval. These statistics were not provided in the original paper*.

Nock (2007) describes the components of psychological interventions for behavior change (Table 2). In short, behavior change is brought about by the mechanisms of change of the intervention, and in psychological interventions, these mechanisms of change are psychological processes. These processes are caused by active ingredients, and the active ingredients consist of specific clinician and client factors and their interaction.

**Table 2:** Components of psychological interventions.

**Clinician factors:** “what the clinician does in the treatment, including clinician behaviors, characteristics, and directives” (Nock, 2007, p. 8s).
**Client factors:** “what the client does in treatment, including behaviors, characteristics, and verbalizations on their part” (Nock, 2007, p. 8s).
**Mechanisms of change:** “the processes that emerge from or occur as a result of the clinician and client factors, and their interaction, that explain how those factors lead to change in the outcomes of interest” (Nock, 2007, p. 8s).
**Active ingredients:** “specific components that cause the observed change” (Nock, 2007, p. 8s).

**Figure 1:**
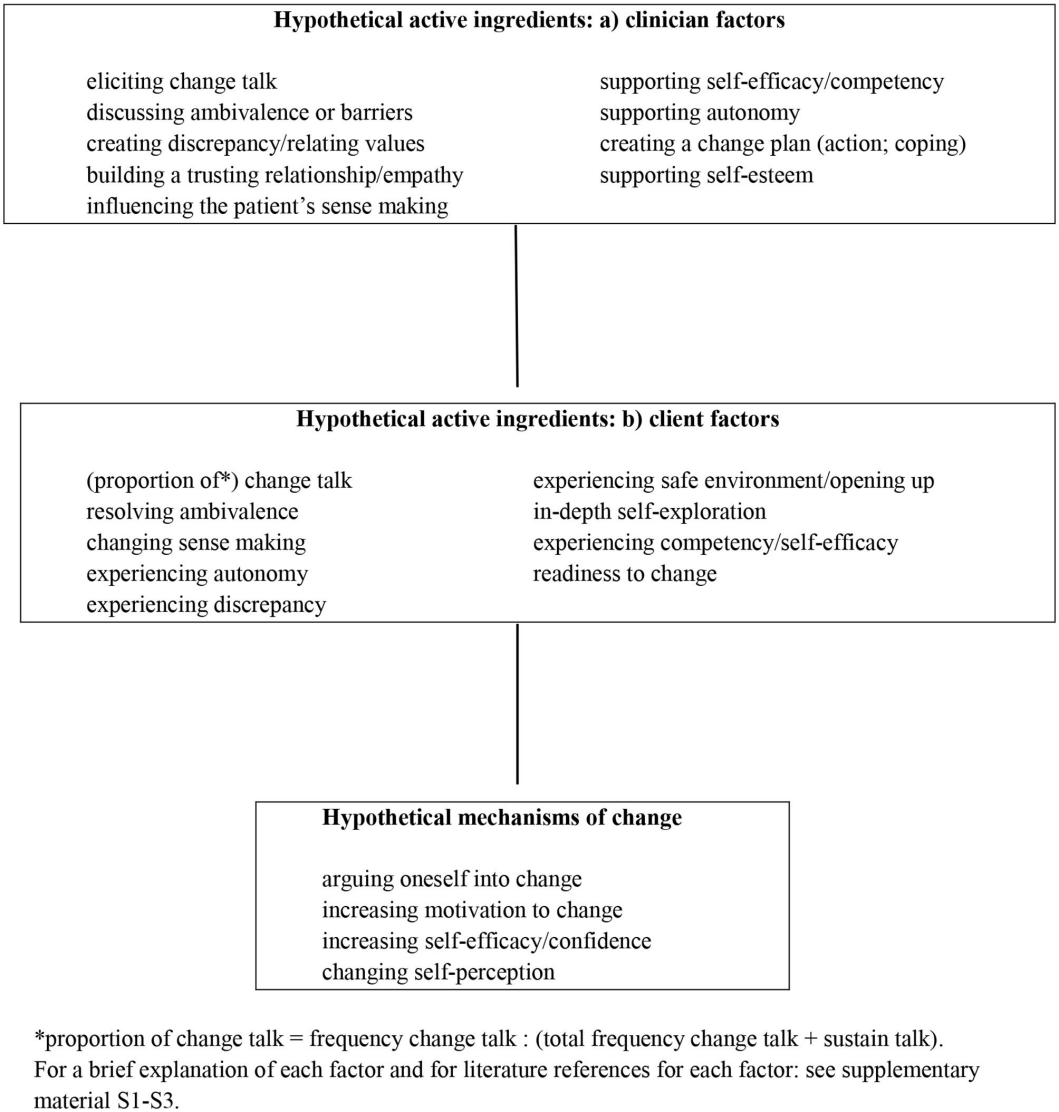
Model of hypothetical active ingredients and mechanisms of change in MI.

The active ingredients and the mechanisms of change of MI are not clear (Miller and Rollnick, 2014). The presence or absence of these ingredients and mechanisms in the intervention content will probably strongly determine the success of the intervention. There are a number of candidate active ingredients (e.g., “change talk”) and candidate mechanisms of change, (e.g., “arguing oneself into change”) (Bem, 1967; Miller, 1983; Gollwitzer, 1999; Miller and Rollnick, 2002, 2004, 2013; McNally et al., 2005; Moos, 2007; Arkowitz et al., 2008; Gaume et al., 2008; Apodaca and Longabaugh, 2009; Miller and Rose, 2009; Barnett et al., 2010; Glynn and Moyers, 2010; Lee et al., 2010; Berger and Villaume, 2013; Moyers and Miller, 2013; Apodaca et al., 2014; Berger and Bertram, 2015; Copeland et al., 2015; Magill et al., 2015, 2018). In a previous study (Dobber et al., 2020), we performed a systematic literature search for active ingredients and mechanisms of change in PsychInfo, in PubMed, and in textbooks on MI. Based on the research (Gollwitzer, 1999; McNally et al., 2005; Gaume et al., 2008; Apodaca and Longabaugh, 2009; Barnett et al., 2010; Glynn and Moyers, 2010; Lee et al., 2010; Apodaca et al., 2014; Copeland et al., 2015; Magill et al., 2018) and MI theory (Bem, 1967; Miller, 1983; Gollwitzer, 1999; Miller and Rollnick, 2002, 2004, 2013; McNally et al., 2005; Moos, 2007; Arkowitz et al., 2008; Gaume et al., 2008; Apodaca and Longabaugh, 2009; Miller and Rose, 2009; Barnett et al., 2010; Glynn and Moyers, 2010; Lee et al., 2010; Berger and Villaume, 2013; Moyers and Miller, 2013; Apodaca et al., 2014; Berger and Bertram, 2015; Copeland et al., 2015; Magill et al., 2015, 2018), we developed a model of hypothetical active ingredients and hypothetical mechanisms of change in the process of MI [see (Dobber et al., 2020) for more details]. In the current study, we used this model (Figure 1) to explore coaching strategies and the use of active ingredients to trigger mechanisms of change in MI for smoking cessation in patients with CAD. The current study aimed to explore active ingredients and mechanisms of change in MI coaching for smoking cessation in patients with CAD, shortly after they had experienced a cardiac event. Thus, we focused on the following questions: (1) Which clinician factors do the coach use? (2) Which client factors are activated by the clinician factors? (3) Does the interaction of clinician factors and client factors lead to a mechanism of change? and (4) Is MI quality related to the use of active ingredients?

## Materials and Methods

### Study Design

We performed a mixed methods study to investigate the strategies of MI coaches in coaching patients for smoking cessation after ACS and/or coronary revascularization. First, we conducted a multiple case study analysis (Stake, 2006) to investigate if and how active ingredients and mechanisms of change appeared in the process of MI. This analysis includes the following three phases: single-case analysis, cross-case analysis, and cross-case synthesis (Stake, 2006). It is an inductive interpretative case study designed to obtain the understanding of psychological processes during coach–patient interaction to promote motivation for smoking cessation. Second, we applied sequential analysis (Bakeman and Quera, 2011) to estimate the probabilities that the application of specific MI techniques by a coach (e.g., a complex reflection on the reasons of the patient for smoking cessation) is immediately followed by a particular patient reaction (e.g., change talk). The Standards for Reporting Qualitative Research (SRQR) checklist (O'Brien et al., 2014) was used for reporting.

### Study Population

The cases were derived from the intervention group of the Randomized Evaluation of Secondary Prevention by Outpatient Nurse SpEcialists-2 (RESPONSE-2) trial) (Minneboo et al., 2017). In RESPONSE-2, the effect of referral to comprehensive community-based lifestyle programs in combination with care as usual, was evaluated for lifestyle change in patients after ACS and/or revascularization. In the RESPONSE2-trial, patients were included up to 8 weeks after hospitalization, if they had at least one of the following lifestyle risk factors: BMI > 27 kg/m^2^, self-reported physical inactivity, self-reported smoking <6 months before hospital admission (see the study report (Minneboo et al., 2017) for exact inclusion and exclusion criteria). One of these lifestyle programs was an MI-based telephone coaching intervention for smoking cessation, performed by the coaches of Luchtsignaal®. Patients who reported to be smokers in the 6 months period before hospital admission were offered the option to enter this program. The Luchtsignaal intervention consists of a maximum of seven MI-based counseling sessions, in a timeframe of 3–4 months, to coach the patient to stop smoking and to persist in not smoking. The Luchtsignaal coaches used an MI-coaching manual, which describes suggestions for the content of the MI sessions. The manual suggests that the first session should last about 30 min, and the follow-up sessions about 15 min. However, the MI coaches are encouraged to tune the content and the session duration to the individual patient process. The four Luchtsignaal coaches (two psychologists, an anthropologist, and a social worker) were experienced MI coaches, trained by certified MI trainers. Since we wanted to analyze the MI process within and across sessions, we set three available sessions as the minimum to observe this process. Consequently, we included cases if at least three sessions had been audio recorded.

Written informed consent was obtained from all patients at entry in the RESPONSE-2 study. This included consent to record conversations. The investigation was approved by the Medical Ethics Committee (Amsterdam UMC, location AMC, number NL41645.018.12).

### Analysis

#### Coding Methods

For coding, we used the motivational interviewing skill code (MISC 2.0/2.1) (Miller et al., 2003, 2008) and the motivational interviewing sequential code for observing process exchanges (SCOPE) (Martin et al., 2005) as the optimal instruments to identify active ingredients of MI (Dobber et al., 2015). The audio recordings were transcribed and subsequently parsed in coach and patient utterances in accordance with the coding manuals (Miller et al., 2003, 2008; Martin et al., 2005). Using both the audio recordings and the transcripts, we coded the relational ingredients of MI (7-point global ratings of acceptance, empathy, and MI spirit) and the sequential verbal interactions between the coach and the patient. Moreover, we scored the MISC global rating for the level of patient self-exploration (Table 3). Based on the premise that the level of patient self-exploration strongly depends on the safety of the coach–patient relationship, we considered a score of >4 on this 7-point scale as an indication of a trusting relationship. To assess the fidelity and the quality of the delivered MI (Martin et al., 2005; Miller et al., 2008; Moyers et al., 2015), we computed summary scores for each session and for all sessions per case. Four summary scores show the extent to which the coach used MI-consistent techniques and the core motivational interviewing skills, the fifth summary score shows the performance of the coaches on the relational component of MI (Martin et al., 2005; Miller et al., 2008; Moyers et al., 2015).

**Table 3:** Measurement of coach and patient behavior.

**Measurement**	**Combined categories for the calculation of the transition probabilities**
*Measurements of coach behavior* Global ratings (Miller et al., 2008): • Acceptance, Empathy, MI Spirit	
Verbal behavior codes (Miller et al., 2008): • advise with permission, advise without permission, affirm, confront, direct, emphasize control, facilitate, feedback, filler, general information, opinion, permission seeking, question, raise concern, reflect, self-disclosure, structure, support, warn, and not encodable	Verbal behavior codes (Miller and Rollnick, 2002, 2013; Moyers et al., 2009): • sMico (affirmation, emphasize control, permission seeking, support) • MIIN (confront, direct, warn, opinion, advise without permission) • Other (facilitate, filler, self-disclosure, general information, raise concern, structure, advise with permission, and not encodable coach statements) • not combined were question and reflect
Summary scores (Martin et al., 2005; Miller et al., 2008; Moyers et al., 2010): • ratio of reflections to questions, percent open questions, percent complex reflections, percent MI-consistent techniques, and mean global ratings	
*Measurements of patient behavior* Global rating (Miller et al., 2008): • level of client self-exploration	
Verbal behavior codes (Miller et al., 2003; Martin et al., 2005): • ask, follow/neutral, commitment, reasons (including desire, ability, and need), taking steps, other, and not encodable	Verbal behavior codes (Miller and Rollnick, 2002, 2013; Miller et al., 2003; Moyers et al., 2009): • Change talk: [reasons (including desire, ability, need) commitment, taking steps, and other pro-change statements] • Sustain talk [reasons (including desire, ability, need), commitment, taking steps, and other counter-change statements] • Neutral (ask, follow/neutral, and not encodable patient statements)

*sMico, sequential MI-consistent behavior; MIIN, MI-inconsistent behavior*.

The first author (JD) received MISC training at the MI-coding lab of the Center for Alcohol and Addiction Studies, Brown University, USA. He trained three coders (two master level, one bachelor level) for coding in the present study (37 h training each). The coders coded the transcript in two passes. First, the coders listened uninterruptedly to the complete session and assigned the global ratings. Second, the coders again listened to the complete audio recording but could pause and listen again to fragments of the conversation to code each parse in one of the coding categories (Table 3). In weekly coder–trainer meetings, we discussed and solved any coding dilemmas. We recoded a random selection of 10% of the sessions (11 sessions) to assess intra-rater agreement, and we independently double coded a random selection of 20% of the sessions (22 sessions) for inter-rater agreement (intra-rater agreement: Kappa behavior codes = 0.80; inter-rater agreement: Kappa behavior codes = 0.82). On the global ratings, a difference of zero or one point on the 7-point scales was considered as agreement or disagreement, if otherwise. Thus, the scores were dichotomized as “agreement” and “disagreement” (intra-rater agreement: Kappa global ratings = 1.0; inter-rater agreement: Kappa global ratings = 0.97).

#### Multiple Case Analysis

All audio-recorded conversations and the coded transcripts of the included patients formed the cases in the multiple case study. Through the multiple case analysis, the analyst (JD) kept a log with detailed information on the research process, the findings, and the decisions. Additionally, to analyze the case and to systematically gather data on how MI coaches applied the main MI components in their coaching, the analyst used worksheets with targets of MI consistency (Allison et al., 2012), organized in accordance with the four MI processes (engaging, focusing, evoking, and planning). We added the concept “sense making” (Berger and Villaume, 2013), which refers to the cognitions and beliefs of the patient about (not) smoking and about the relationship between smoking and the heart condition of patients. Berger and Villaume (Berger and Villaume, 2013) integrated this concept into MI to help the coaches understand the perspective of the patient, and enable the coach to more effectively apply the clinician factors. This worksheet provided insight on how in each case, coaches applied the clinician factors to activate specific client factors (Figure 1). Further, we composed a second worksheet based on our model of hypothetical active ingredients and mechanisms of change. For each case, we composed a detailed case report. Two co-authors (CL and BvM) verified these steps and checked the decisions made during data analysis, the integrity of the findings, and the conclusions, and another co-author (MS) independently double analyzed two cases to check the repeatability of the findings. In case of disagreements, we checked the original data to resolve the disagreement. All coders and all persons involved in the qualitative analysis were blinded for the outcome “smoking status at 12 months after baseline.”

#### Transition Probabilities

Furthermore, using Generalized Sequential Querier software (GSEQ 5.1) (Bakeman and Quera, 2011; GSEQ 5.1), we calculated the probabilities that specific MI-conversational techniques were immediately followed by patient change talk or patient sustain talk of the patient. For example, what is the probability that the use of a reflection directed at the advantages of smoking cessation by the coaches was immediately followed by the change talk of the patient. Hereto, the GSEQ 5.1 combines all patient statements of all MI sessions in one pool, and all coach statements in a second pool. Thus, these probabilities are calculated on the basis of all coach statements and the immediately following patient statements across all sessions. Due to the low occurrence in our sample of some verbal behavior codes, we combined these behaviors in line with MI theory (Miller and Rollnick, 2002, 2013) and with a previous study (Moyers et al., 2009) (Table 3).

#### Smoking Status

In the RESPONSE-2 trial (Minneboo et al., 2017), from which the cases originate, the smoking status of the patients was assessed at baseline and after 12 months, using a urinary cotinine test (UltiMed one step, Dutch Diagnostic, Zutphen, the Netherlands; detection limit 200 ng/ml). Finally, based on an emerging pattern of smoking cessation in cases in which one or more of the mechanisms of change in MI were observed, we calculated the risk ratio *post hoc* to verify this possible association.

## Results

We included 24 cases, of which at least three MI sessions were audio recorded, out of the 50 patients that took part in the Luchtsignaal intervention, comprising 109/151 (72.2%) MI sessions. Of the 24 patients, 16 patients completed all 7 MI sessions of the intervention, while 8 patients did not complete the intervention. In seven cases, all sessions were recorded. There are five cases missing one recorded session, four cases missing two recorded sessions, three cases missing three recorded sessions, and five cases missing four recorded sessions. Table 4 shows the baseline characteristics of the participants. One coach performed MI in 72 sessions, one in 23 sessions, one in 11 sessions, and one coach performed MI in 3 sessions.

**Table 4:** Baseline characteristics^a^.

	**Luchtsignaal-intervention: cases in this analysis (*n* = 23^b^)**
**Demographics**	
• Mean age (range) • Female • Caucasian • Higher education (>13 years) • Relationship (married or cohabiting)	55.4 (36– 75) 4 (17.4%) 21 (91.3%) 6 (26.1%) 17 (73.9%)
**Treatment**	
• Percutaneous coronary intervention • Coronary artery bypass surgery • Medication only	17 (73.9%) 2 (8.7%) 4 (17.4%)
**Smoking status**	
• Smoking at baseline • Recent quitters (<6 months prior to baseline)	18 (78.3%) 5 (21.7%)
**Lifestyle-related risk factors**	
• Smoking only • Smoking and BMI^c^ > 27 • Smoking and physical inactivity • Smoking and BMI > 27 and physical inactivity	4 (17.4%) 2 (8.7%) 5 (21.7%) 12 (52.2%)
**Medication**	
• Antiplatelet agents • Beta-blockers • ACE inhibiter/ARB^d^ • Lipid-lowering drugs	23 (100%) 15 (65.2%) 14 (60.9%) 21 (91.3%)

a *Baseline measurement of the RESPONSE2-intervention. Often, the MI intervention for smoking cessation started a few weeks or months after the measurement of the RESPONSE2-baseline measurements*.

b *The baseline characteristics of one patient are missing due to the use of an incorrect patient identification number by Luchtsignaal*.

c *BMI, Body mass index*.

d *ACE, Angiotensine Converting Enzyme; ARB, Angiotensin Receptor Blockers*.

Five patients used NRT, seven used varenicline (Champix®), and twelve patients, mainly the patients who had already reduced their smoking, did not use either of these therapies (Table 5). At the start of the MI sessions, 4 out of 24 patients (16.7%) were self-reported to be non-smoking since the cardiac event. The difference between the number of self-reported non-smoking and the number of recent quitters in the baseline characteristics in Table 4 arises from the elapsed time—often a few weeks or months—between the randomization in the RCT and start of the MI intervention for smoking cessation. At the end of the last recorded MI session (note: not all last sessions were audio recorded), 16 out of 24 patients self-reported to be a non-smoker (66.7%; Table 5). Twelve months after baseline, 10 out of 21 patients had a negative urine cotinine test (<200 ng/ml), indicating that they were non-smokers (47.6%; three missing values). Only four patients using NRT or varenicline were non-smokers at 12 months, seven of these patients were smokers, and there was one missing value (Table 5). The most prevalent motive for smoking cessation was “prevention of another myocardial infarction and other health issues,” while “stress” was the main reason to continue smoking (Table 6).

**Table 5:** Characteristics of smoking behavior and ambivalence.

**Number of cigarettes/ cigars per day at the start of MI (*n*)**	**Number of cigarettes/cigars per day at the last audio-recorded MI session^a^ (*n*)**	**Smoking status at 12 months smoker/non-smoker^b^ (*n*)**	**Medication (*n*)**	**Ambivalent about (*n*)**
0^c^ (4)	0 (4)	S (1)N (2)- (1)	None (4)	Persistence (ability) (4)
<1 (2)	0 (1)<1 (1)	N (1) S (1)	None (1) None (1)	Ability to quit (2)
2–5 (3)	0 (2)	S (1)N (1)	None (1), NRT (1)	Ability to quit (2)
	<1 (1)	S (1)	None (1)	Ability to quit (1)
6–10 (4)	0 (1)	- (1)	None (1)	Willingness and ability to quit (1)
	2–5 (2)	S (2)	NRT (1), varenicl^c^ (1)	Ability to quit (2)
	6–10 (1)	N (1)	None (1)	Ability to quit (1)
11–15 (1)	0 (1)	N (1)	None (1)	Ability to quit (1)
>16 (5)	0 (2)	S (2)	NRT (2)	Ability to quit (2)
	2–5 (2)	S (2)	Varenicl^d^ (2)	Ability to quit (1)
				Willingness and ability to quit (1)
	11–15 (1)	- (1)	Varenicl^d^ (1)	Ability to quit (1)
Unclear (5)	0 (5)	S (1)	NRT (1)	Ability to quit (1)
		N (4)	None (1) varenicl^d^ (3)	Ability to quit (4)

a *In some cases, the last MI session was not recorded*.

b *Smoking status (based on urine cotinine test) 12 months after baseline of the original RESPONSE-2 trial (Minneboo et al., 2017). Often, the MI-intervention cessation started a few weeks or months after the measurement of the RESPONSE-2 baseline measurements. S, Smoker; N, Non-smoker; -, missing value*.

c *The four patients that reported non-smoking at the start of MI are recent quitters. They stopped smoking after the cardiac event and felt ambivalent about their ability to persist in non-smoking*.

d *Varenicl, varenicline*.

**Table 6:** Motives for (not) smoking.

**Motives for smoking cessation (frequency)** • The myocardial infarction (13) and other health-related motives (24) • For my children (7), partner (5), grandchildren (3) • Improves my quality of life (10) • Saves money (5) • Smoking stinks (4) • Not allowed to smoke anywhere anymore (4) • To live longer (3) • Other motives^*^ (16)	**Motives for smoking (frequency)** • Stress (9) • Need for nicotine/dependency (4) • Habit/automatism (3) • Other motives^*^ (12)

* *Each other motive was only mentioned once or twice*.

In the sections below, we will first discuss the occurrence of clinician factors and client factors in the MI sessions. These clinician factors and client factors may interact such that they become active ingredients and trigger a mechanism of change (these mechanisms may explain how patients decide to quit smoking) (Nock, 2007). Second, we describe the occurrence of mechanisms of change in the MI sessions. Our model of hypothetical active ingredients and mechanisms of change (Figure 1) helps us to concentrate on the relevant factors and mechanisms. Thus, this part of the “Results” section deals with the question of *whether these factors and mechanisms occur* in the MI sessions. The next part of the “Results” section is about *how the MI coach applies these factors* to stimulate active ingredients and mechanisms of change. Hereto, we first describe the use of MI-conversational techniques and then the MI strategy of the coaches in the four MI processes (engaging, focussing, evoking, and planning). Finally, we will relate the occurrence of active ingredients to delivered MI quality.

### Clinician Factors

Taking all 109 sessions and all four coaches together, the coaches applied all clinician factors (Figure 1), except “creating a change plan” (Table 7). So, although “the how and when of smoking cessation” often was discussed, the patients and their coaches failed to compose concrete change plans. The number of different clinician factors applied varied among the cases. The mean number of different client factors over 24 cases is 4.38, with a range of 3–6. This indicates that the lowest number of different clinician factors in a case is 3, and the highest number of these factors is 6. With all patients, the coaches established a trusting relationship, as indicated by a score of >4 on the 7-point global rating for the level of patient self-exploration. The empathy of the coach was a crucial factor in establishing this relationship. In line with MI theory, the clinician factor “eliciting change talk” was used most frequently. After this, “supporting self-efficacy” and “discussing ambivalence or barriers” were the most frequently applied clinician factors. This is consistent with our finding that the ambivalence that most patients experienced was about the ability to stop smoking (Table 5).

**Table 7:** Hypothetical active ingredients (clinician factors and client factors), and mechanisms of change in 109 MI sessions.

**Hypothetical clinician factors**	**Freq**	**Hypothetical client factors**	**Freq**	**Hypothetical mechanisms of change**	**Freq**
Building a trusting relationship/empathy	^*^	Experiencing safe environment/opening up	^*^		
Eliciting change talk	210	Change talk	198	Arguing oneself into change	10
				Increasing self-efficacy/confidence	3
				Increasing motivation to change	3
		Sustain talk^**^	51		
		In-depth self-exploration	4		
		Readiness to change	1		
		Experiencing autonomy	1		
		Changing sense making	2		
		Resolving ambivalence	1		
		Experiencing self-efficacy/competency	3		
Creating discrepancy/ relating values	3	Experiencing discrepancy	1		
		Change talk	1		
Discussing ambivalence or barriers	51	Resolving ambivalence	1		
		Change talk	39		
		Sustain talk^**^	36		
		Experiencing discrepancy	8		
		Experiencing self-efficacy/competency	5		
		In-depth self-exploration	6	Arguing oneself into change	1
		Readiness to change	1	Arguing oneself into change	1
Influencing the sense making of the patient	6	Changing sense making	3	Arguing oneself into change	1
Supporting self-efficacy/competency	116	Experiencing self-efficacy/competency	92	Increasing self-efficacy/confidence	10
				Arguing oneself into change	2
		Change talk	8	Arguing oneself into change	1
		Sustain talk^**^	7		
		Experiencing autonomy	2		
		In-depth self-exploration	1	Arguing oneself into change	1
Supporting self-esteem	3	Change talk	1		
Supporting autonomy	4	Experiencing autonomy	4		
		Change talk	1		
Creating a change plan	0				

* *mostly applied and maintained through all sessions*.

** *sustain talk is a client factor in favor of persistent smoking*.

### Client Factors

All nine client factors included in our model (Figure 1) appeared in the sessions, with a mean of 4.08 different client factors per patient (range 2–7), and with “(proportion of) change talk” as the most prevalent one (Table 7). Often, clinician factors activated two or more client factors simultaneously. “Supporting self-efficacy,” for instance, activated both “experiencing self-efficacy/competency” and “change talk.” The application of clinician factors was quite successful, as these factors nearly always activated client factors. Considering the dominant type of ambivalence (willing to stop, but perceiving the inability to quit smoking), the coaches needed to activate the client factor “experiencing self-efficacy/competency.” This client factor was activated by the clinician factors “eliciting change talk,” “discussing ambivalence or barriers,” and mostly by “supporting self-efficacy/competency” (Table 7).

### Mechanisms of Change

The mechanisms of change of MI are psychological processes, so they take place in the mind of the patient. As a consequence, these mechanisms of change cannot be observed directly. So, we restricted ourselves, on the basis of the speech of the patient, to the recognition of clues indicating the appearance of these psychological processes. Across the 109 sessions, we identified 33 of these clues, which were recognized in 12 of the 24 patients (mean number observed over 12 patients was 2.75, range 1–7). Seventeen times, we observed clues for “arguing oneself into change” (see Box 1 for an example), thirteen times for “increasing self-efficacy/confidence,” and three times for “increasing motivation to change.” We did not find clues for “changing self-perception” (Table 7). Mechanisms of change were mostly preceded by an interaction between a variety of clinician factors and client factors.

Box 1 **Example of a clue for the mechanism of change “arguing oneself into change.”**

*Case 22, fragments of session 2*

(…)

Patient: “Smoking can cause a lot of damage, like yesterday on television, there was a man with something on his heart valves. And he was… it affects me, it makes you think this could also have happened you know.”

Coach: “Yes, what could have happened?”

Patient: “He smokes too and things like that. It affected me, makes me think…”

Coach: “What could have happened…”

Patient: “Yes, what could have happened. Like the phone, his phone was downstairs and not beside his bed, so he had to go downstairs, but shortage of breath and things like that, well you know. It scared me, made me shed a tear.”

Coach: “It could have been me.”

Patient. “Yes, I didn't experience this, but if you're upstairs and your telephone is downstairs, what can happen in between? And no breath.”

Coach: “What made it so emotional for you?”

Patient: “That something like this may happen, abruptly. Well it makes me sympathize with him, I kind of experienced that. Not exactly like him, a little different.”

Coach: “The vulnerability.”

Patient: “Yes, so…”

Coach: “It's a bit like what happened to you.”

Patient: “No, that's why, you have to make decisions like to continue living your old life or start living a new life. Like this is not allowed, and that is not allowed, no, yes, no more smoking for me, but let's say I'll have to stay a bit away from food and tasty things. So, living a healthier life.”

Coach: “It's worth a lot to you actually, it is important for you to stay around.”

Patient: “Yes, I think so, 47, and then… no, please not yet, I still want to live for a while, so…”

Coach: “You're making all sorts of adjustments.”

Patient: “You must, can't go on like this, like I used to do. Or, in a few years, you won't be around anymore.”

(…)

Coach: “So your confidence in not having a cigarette is higher than last time. (…) How did that happen?”

Patient: “How, because I don't need it at all, though sometimes I think about a smoke, but if someone would stand in front of me offering me a cigarette? No, I would not take one.”

Coach: “So when you think about smoking, you're able to handle this quite well, which makes you trust you won't relapse.”

Patient: “No I won't fall back. Well, I'm not 100% sure, but I'm 99% sure that I will not have a cigarette, I won't fall back.”

In 12 cases, we did not observe clues for a mechanism of change. In nine of these cases, not all sessions were audiotaped, and there may have been clues for the mechanisms of change in these missing sessions. In the three cases with audio recordings of all sessions, the coaching was prematurely finished before the intervention had been completed.

### Application of Active Ingredients to Influence Mechanisms of Change

We describe two levels of the applications of the active ingredients of the coaches. The first level is the level of “which conversational techniques do evoke change talk on smoking cessation?” On the second level, we elaborate on the MI strategies of the coaches to trigger the mechanisms of change.

#### Conversational Techniques

In Table 8, the results of the quantitative sequential analysis (GSEQ 5.1; Bakeman and Quera, 2011) are displayed, which was performed over all 109 MI sessions to calculate the transition probabilities, i.e., the chances that a certain type of patient statement (sustain talk, change talk, and neutral statement) follows directly after a certain type of conversational technique (or coach statement; see Table 7 for types of coach statements). The chance of a client expressing “change talk,” immediately following *a reflection* directed at smoking cessation behavior or intentions (Reflection+), was 76%, and there was a 66% chance of change talk following *a question* directed at the smoking cessation behavior or intentions (Question+). The chance of “sustain talk” was the highest after questions and reflections directed at persisting in smoking (both 58%, Table 8).

**Table 8:** Transition probabilities^a^^b^ of patient statements following a coach statement.

**Target (patient statements; *n* = 8,307)Given (coach statements; *n* = 7,340)**	**Sustain talk (freq. = 1,308)**	**Change talk (freq. = 3,234)**	**Neutral (freq. = 3,765)**	**Frequency**
Other^c^	0.06	0.16	0.78	2,241
two-sided question (±)^d^	0.21	0.52	0.27	338
Question-^d^	0.58	0.12	0.30	306
Question neutral^d^	0.03	0.08	0.89	592
Question+^d^	0.05	0.66	0.29	526
two-sided reflection (±)^d^	0.21^†^	0.43^†^	0.36^*^	171
Reflection-^d^	0.58	0.16	0.25	542
Reflection neutral^d^	0.05	0.09	0.86	487
Reflection+^d^	0.05	0.76	0.20	1,154
sMI-consistent^e^	0.03	0.19	0.78	767
MI-inconsistent^f^	0.07	0.37^†^	0.56	216

a *Probability of a certain type of patient statement immediately following a particular type of coach statement (lag = 1)*.

b *All: p < 0.01, except*

* *p = 0.03 and*

† *not significant*.

c *Other comprises facilitate, filler, self-disclosure, general information, raise concern, structure, advising with permission, not encodable*.

d *Two-sided means questions or reflections addressing both change talk and sustain talk; + means questions or reflections directed at smoking cessation behavior or intentions; − means questions or reflections directed at persistent smoking behavior or intentions; neutral means questions or reflections directed at other topics than smoking behavior*.

e *sMI-consistent, sequential MI-consistent, and comprises affirmation, emphasizing control, permission seeking, and offering support*.

f *MI-inconsistent comprises confrontation, directing, warning, giving opinion, and advising without permission*.

#### MI Strategy

To provide insight on the MI strategy of the coaches, we describe how the MI coaches applied the active ingredients in the MI-processes: engaging, focusing, evoking, planning.

### Engaging: Establishing a Trusting Relationship

The clinician factor “building a trusting relationship” usually happened in the first part of the first session, and sometimes in the second session. The MI coach showed interest in the experiences of the patient after the ACS or the revascularization, and this active listening with empathetic reflections seemed to form the basis for the trusting relationship. A superficial acquaintance or taking up the expert role hindered the development of a trusting relationship at the cost of less depth in the conversations. See Box 2 for an example of a good trusting relationship. The coach persisted in talking about a subject the patient preferred to avoid, without causing friction.

Box 2 **Good trusting relationship.**

*Case 6, seventh (last) session (3 months ago, after the first session, the patient stopped smoking)*

The coach starts the session by asking how things are going, and the patient answers, “I'm doing fine, actually,” and continues talking about her holidays. The coach takes the subject back to smoking:

Coach: “You're doing fine, but last time you said: ‘It's going very well.’ Is that a coincidence, or is it something…?”

Patient: “No, it has not really changed specifically.”

Coach: “No, how would you describe more precisely how things are going now?”

Patient: “Well, if you take it specifically back to not smoking, I may be not as entirely motivated as I was at the start. Very occasionally, I took a cigarette puff. Incidentally, really incidentally, but I did. But since last week, I think, I have not smoked anymore, it doesn't bother me.”

Coach: “The motivation dips a bit.”

Patient: “Well not really, yes the motivation, there may have been moments, and I can't say why, that I longed for a cigarette, and eventually, I had a puff. But I don't think it takes a lot of effort not to. So, I say no, it's not an effort not to.”

Coach: “Is it also an intention not to, or has it become an intention to ‘rather not, but if I long for it, I'll have a puff’?”

Patient: “No, I quit. Definitely quit. Actually, that time I smoked a bit, it didn't really do something for me, give me something. So, I mean, right now I've stopped. I feel I've stopped.”

(…)

*The conversation continues why these puffs may have happened. The coach asks the patient to indicate how she perceives the importance of not smoking as a mark on a ruler from 1 to 10*.

Patient: “Definitely a high number, (…) a 7, 8, or 9? I don't know.”

Coach: “In the first session it was a 9.”

Patient: “Okay, I see. Well you wrote it down and I don't remember. And that's okay, I know. But yes, the motivation is there, and like I said, I feel I have stopped.”

Coach: “That's why you're less concerned with the why of smoking cessation.”

Patient: “I also put on some weight, my weight shows that I stopped smoking.”

Coach: “You're not smoking. (…) Coming back to your motivation, what are the main reasons for a high number? Regardless of the exact number. What are the things that you think ‘yes, that is why I stopped smoking.’?”

(The patient responds talking about her motives for smoking cessation.)

Another result of the engagement process is the information on the sense making of smoking by the patient. An example is a patient who, after her myocardial infarction, stopped smoking, but, every now and then, she needed to take a puff of a cigarette. She called the cigarette a “pal,” helping her with stress, to sit apart and enjoy the cigarette. The myocardial infarction abruptly ended this relationship, without a proper goodbye (Case 14). This kind of knowledge of the sense making of the patient allowed the coach to choose clinician factors that connected with the cognitions of the patient, and these clinician factors might influence the way of thinking of the patient about smoking. The clinician factors, chosen by the coach based on the sense making of the patient, might also allow the coach to relate smoking with the life goals and values of the patient (see Box 3 for a more extensive elaboration; see Table 9 for more examples of cognitions about smoking).

Box 3 **Influencing patient sense making; fragments of good MI.**

**Table:** 

	**Comments**
*Case 14* *This patient stopped smoking after her myocardial infarction. But every now and then she needs to take a puff of a cigarette*.	
*Session 1* (…)	
Patient: “I kind of would like to smoke my last cigarette you know, like I never renounced it (…).”	
Coach: “Like I'm not there yet, not completely done yet. I did stop but I didn't break the bond with smoking.”	Reflection, active listening, and empathy
Patient: “Yes. Maybe so, yes. (…) I already had the intention to quit, but had to stop having that heart attack. And I did. But then and now in my mind, like I decide when I smoke the last one and after that I'll never smoke again. I didn't have that.”	Need for autonomy, and safe environment/ opening up
Coach: So the final goodbye has yet to take place.”	Reflection
Patient: “That's how I feel, yes.”	
(…) *The patient and the coach review the motives of the patient regarding smoking cessation*.	
Coach: “Many cons, hardly any pro. Still, you smoked and you wanted to say goodbye to your cigarettes. Apparently smoking did something for you.”	Reflection of the ambivalence
Patient: “Yes, it did, I considered it as a pal. It was good for me when I had stress. To sit apart and enjoy my smoke. And probably… it made me feel relaxed. It wasn't an enemy, it was kind off a friend.”	Sustain talk revealing the sense making of the patient about smoking
Coach: “You had those moments together. Especially when life was a little tough, like stress, retreat with a cigarette.”	Reflection, Empathy
Patient: “I did, I did.”	
(…) *The coach provides the patient with information on the working mechanism of nicotine on blood vessels, and on the risk of another heart attack, and the coach emphasizes the control of the patient: it is her decision*.	Influencing the sense making of the patient, Supporting autonomy
Patient: “I see what you mean. (…) It has been a friend in my mind, but not for my body, I see what you mean, you don't say goodbye to a good thing.”	Change talk
Coach: “It had two sides, like a bad friend. Like parents sometimes say about their children's friends, there are good friends, but also bad friends. You can also have a good time with them, but they might get you in trouble. Parents see that they're no good to their children. A bad friend, no enemy, but a bad friend. Like you say: bad for my body, but we had some good times.”	Reflection, Influencing the sense making of the patient
Patient: “Yes, I see what you mean, yes. (…) Right now I'm thinking yes, in my mind there were good moments, but on the other hand, it ruined a lot.”	Change talk, Changing sense making, Arguing herself into change
*Session 2* (…)	
Patient: “It was my own decision, it wasn't a complete one, just a few puffs, a half one. It was a good thing to do I think.”	
Coach: “And what did you think and feel?”	Evoking
Patient: “It didn't taste good, it ruins a lot, I realize it does. (…) It is closure. I'm glad I did this, it would stick in my mind.”	Change talk, Commitment
Coach: “(…) it actually gave you space: I could put an end to it, it is not so important for me anymore.”	Reflection
Patient: “Yes, you keep searching, the searching is now kind of gone.”	Resolving ambivalence, Arguing herself into change
*Session 4* (…)	
Patient: “I've moved on after our first conversation. It's the notion that it ruins a lot. (…) That's the standard now, just don't smoke anymore. Just keep that image in mind.”	Change talk, Commitment
*Session 5* (…)	
Patient: “I've come to this point that I'll never smoke again, one hundred percent.”	Change talk, Commitment
Coach: “(…) So what made you move since our first conversation?”	Evoking to strengthen change talk/commitment
Patient: “I kept thinking about it, every time I walk into it I find it filthy, it really stinks, but most of all what you said the first time, it really hit me. Because first, it was a friend, and you followed me in that. But then, I realized what you really said, it ruins your body, it's not much of a friend. And often, this goes through my mind, I tell that to others too, yes.”	Change talk

**Table 9:** Examples of cognitions on smoking.

• “The dependency, the fact that I can't leave home without my cigarettes (…) I'm walking on the beach with cigarettes in my swimsuit… so stupid!” • “Smoking is an addiction, it will never pass, but I want to be in control” • “Smoking is an automaticity, more and more often I catch myself with a cigarette in my hands” • “Without a cigarette, I can't really wake up” • “I need nicotine in the morning, I need a cigarette when I wake up. To start up” • “Sometimes I think what does it matter, a cigarette more or less (…) I don't live in a busy city with traffic constantly passing by, I live in a clean village” • “It is nonsense to blame it all on smoking. Smokers can become very old, non-smokers can also have a heart condition”

### Focusing: Concentrating on the Change Goal

In this process, the coach ensures the change goal to be central to the conversation. In 83% of the sessions, coaches kept the conversation on target. We observed weak guidance from the coach in 19 sessions, which was expressed in superficial conversations with a question-answer pattern, off-target chat, a lack of complex reflections, giving too much information and advice not linked to the concerns of the patient, focusing on importance while the concern of the patient was about ability, or vice versa. The effect of weak guidance was visible in a lack of effective use of clinician factors, resulting in the absence of relevant client factors and a lack of progress in the thought processes of the patient on smoking cessation.

### Evoking: Helping the Patient to Find and Voice His/Her Own Motives

Evoking and strengthening the motivations of the patient for change is a fundamental characteristic of MI (Miller and Rollnick, 2014). The quality of the evocation in the cases varied from very good (4 cases) (see Box 4a for an example), good (4 cases), sufficient (11 cases), to insufficient (5 cases). A weak evocation style was characterized by hardly elaborating on change talk, asking too many questions, and offering too few reflections (see Box 4b for an example), resulting in a failure to activate relevant client factors. In some cases, the insufficient evocation seemed to be related to the needs and choices of the patient, e.g., one patient wanted to find in-depth psychological reasons for his smoking behavior, and another patient chose to prioritize weight loss as the target behavior instead of smoking cessation.

Box 4 **Examples of good (a) and weak (b) evocation.**

***4a*** *Good evocation*

*Case 7, session 5*

(…)

Coach: “Are there other things, apart from you saying ‘I can smell much better’?”

Patient: “The taste, and less-at long last-less stress and things like that.”

Coach: “You sense that you've calmed…”

Patient: “Yes, like an inner peace. Sometimes I see smoking people, a quick smoke at the door, it doesn't look relaxed, and then I think I don't have that anymore, I'm done with it, finished.”

Coach: “No complicated maneuverings for a quick smoke.”

Patient: “All just for a quick smoke, yes. A few times, when I went for diner with my colleagues, I could remain sitting easily, smoking is no issue anymore. Before, it kept me kind of occupied: when will I stand up and smoke, what is the best moment, things like that. That's over now.”

Coach: “It sort of distracted you, maybe keeping an eye on people, when are they going outside?”

Patient: “Yes.”

Coach: “It is kind of funny that you experience that, isn't it, many people feel that smoking makes them relax, but you say actually it is much more relaxed not to smoke.”

Patient: “Yes. Always, I used to think smoking helps to relax, but that is not true at all, it's the opposite.”

(…)

Coach: Actually, you say, it is pleasant no longer needing to smoke. That is, you kind of started saying like it also has benefits.”

Patient: “Yes, many benefits actually.”

Coach: “Yes, are there other things you…?”

Patient: “Yes, at home, with kids growing up, they also might do this kind of thing. Now, that is much less of a problem, because they know why I've stopped, and they've seen how hard it has been for me. There is less need to try, you know.”

Coach: “Yes, you feel you're a much better role model, the whole picture fits better.”

Patient: “Yes.”

Coach: “Great, good for you!”

_________________________________________________________________________________

***4b*** *Weak evocation*

*Case 2, session 3*

(…)

Coach: “Do you still know why you stopped? Why is it so important?”

Patient: “Yes, yes, my health.”

Coach: “Huh? Your health.”

Patient: “Yes.”

Coach: “Especially your heart.”

Patient: “Yes.”

Most patients related their desire to quit smoking to an important value, namely their health. Sometimes a family member was mentioned as an important value: “I want to stop for my children.” In a few occasions, the coach elaborated on these values and supported the patient to engage in a more in-depth exploration of the relationship between these values and smoking (client factors “in-depth self-exploration” and “experiencing discrepancy”). But often, in situations where the health of the patient was discussed, the coaches tended to give information on the physical effects of smoking, leaving the chance of activating these client factors unused.

Providing the patient with information played an important role in the sessions. Well-timed- and well-provided information can evoke client factors concerning the sense making of, resolving ambivalence of, and change talk of the patient. In MI, it is important to provide only these pieces of information the patient wants and needs. This went well when the coach only provided information if the patient explicitly or implicitly permitted the coach to give this information, after which the coach provided the information in clear language and in small amounts. After providing the information, the coach should inquire after the understanding and interpretation of the information by the patient. The absence of this inquiry about the interpretation by the patient was the most common shortcoming in the information exchange, followed by providing too much information at once and providing unsolicited information. These shortcomings impeded activation of relevant client factors and limited the absorption of the essence of the information by the patient.

### Planning: Developing Commitment to Change, Creating an Action Plan

For all smoking patients, the patient and the coach determined a stop date for smoking, and they planned the prior preparations, including obtaining NRT or varenicline, and, if applicable, the coach provided user instructions for NRT or varenicline. Also, in many sessions, the coaches enquired after difficult (typical) smoking situations, explored these situations, and discussed possible coping strategies. However, in none of the cases, an explicit activity or coping plan was made.

### MI Quality and Application of Active Ingredients

Table 10 shows the number of clinician factors, client factors, clues for the mechanisms of change per case, the actual cotinine verified smoking status of the patient at 12 months after the baseline of the RESPONSE-2 trial, and five summary scores of the performance of the coaches. Based on these summary scores, we assessed each case on well-accepted criteria (Miller et al., 2008; Moyers et al., 2010; Miller and Rollnick, 2014). These summary scores are based on the coding of all available MI sessions per patient and form an indication for the intervention fidelity and the MI quality delivered (Miller and Rollnick, 2014).

**Table 10:** Number of observed clues for mechanisms of change, clinician and client factors, smoking status, and summary scores per case.

**Case (number of audio recorded sessions)**	**Number of clues for mechanisms of change/clinician factors/client factors^a^**	**Smoking status at 12 months Smoker/Non-smoker^b^**	**Summary scores**				
			**Global coach ratings^c^**	**Reflection/ question ratio^d^^,^^e^**	**Proportion open questions of all questions asked^d^^,^^f^**	**Proportion complex reflections of all reflections^d^^,^^g^**	**Proportion MI-consistent behavior^h^**
1 (4)	0/5/4	S	++	+	–	++	+
2 (5)	0/3/3	S	++	–	–	++	+
3 (4)	1/4/3	N	++	++	+	++	+
4 (3)	0/4/3	S	+	+	–	++	+
5 (7)	2/5/4	S	++	+	+	++	+
6 (5)	1/4/5	N	++	+	+	++	+
7 (3)	4/4/4	N	++	+	+	++	++
8 (4)	0/5/5	N	+	+	+	++	+
9 (3)	0/3/2	S	++	+	+	++	+
10 (7)	7/5/5	–	++	+	–	++	+
11 (5)	3/5/3	N	–	++	–	++	+
12 (4)	0/4/3	S	++	++	–	++	+
13 (5)	4/5/7	N	++	+	–	++	+
14 (6)	4/5/7	N	++	++	–	++	+
15 (6)	2/6/5	S	+	–	–	++	+
16 (6)	2/6/4	N	++	+	–	++	+
17 (4)	0/4/4	S	+	+	–	++	+
18 (5)	0/5/5	S	++	+	–	++	+
19 (6)	0/4/4	–	++	+	+	++	+
20 (3)	0/4/5	N	++	+	–	++	+
21 (3)	0/3/3	–	+	+	–	++	+
22 (4)	1/4/4	S	++	+	+	++	+
23 (3)	2/4/3	N	++	+	+	++	++
24 (4)	0/4/3	S	+	++	–	++	+

*Scores are means over all of the audio recorded sessions of the patient*.

*– = not proficient; + = beginning proficiency; ++ = competent (based on thresholds in manuals; Miller et al., 2008; Moyers et al., 2010).*

a *Total number of mechanisms of change during all sessions/number of different clinician factors during all sessions (maximally 9, see Figure 1) /number of different client factors during all sessions (maximally 9, see Figure 1)*.

b *Smoking status (based on urine cotinine test) 12 months after baseline of the original RESPONSE-2 trial (Minneboo et al., 2017). Often, the MI-intervention cessation started a few weeks or months after the measurement of the RESPONSE-2 baseline measurements. S, Smoker; N, Non-smoker; -, missing value*.

c *Global coach ratings show the coach performance on the relational component of MI. Scores are based on mean ratings on three 7-point global rating scales (Acceptance, empathy, MI spirit) (Miller et al., 2008). Threshold beginning proficiency: mean rating = 4.9; threshold competency: mean rating = 5.6 (Moyers et al., 2010)*.

d *These summary scores show the extent to which the coach uses the core MI skills*.

e *Reflection/question ratio. Ratio between reflections and questions (Miller et al., 2008). Threshold beginning proficiency if R: Q = 1; threshold competency if R: Q = 2 (Moyers et al., 2010)*.

f *Proportion open questions of all (open and closed) questions (Miller et al., 2008). Threshold beginning proficiency if % OQ = 50%; threshold competency if % OQ = 70% (Moyers et al., 2010)*.

g *Proportion complex reflections of all (simple and complex) reflections (Miller et al., 2008). Threshold proficiency is % CR = 40%; threshold competency is % CR = 50% (Moyers et al., 2010)*.

h *Proportion of MI-consistent behavior of MI-consistent- and MI-inconsistent behavior (Miller et al., 2008): this score shows the extent to which the coach uses MI-consistent techniques. Threshold beginning proficiency if % MICO = 90%; threshold competency if % MICO = 100% (Moyers et al., 2010)*.

In this small sample, we found an association between the observed clues for the mechanisms of change and the smoking status of the patient at 12 months (Table 10), with the probability to stop smoking increasing from 20 to 72%. We decided *post hoc* to calculate the risk ratio of smoking cessation of patients who had exhibited a mechanism of change and found the risk ratio to be 3.6 (95% CI 0.99–12.22). This can be an indication that the MI quality may be related to the application of active ingredients and, thus, mechanisms of change.

As shown in the columns of the summary scores (Table 10: columns 4–8), coaches asked more closed questions than open questions in the majority of the cases, but they performed very well in offering complex reflections (i.e., reflections that add meaning or emphasis to what the patient has said; Miller and Rollnick, 2013). Based on the summary scores, the quality of the MI delivered was sufficient to good, except for three cases, in which the MI quality was insufficient (Cases 2, 11, and 15).

We observed many clues for mechanisms of change, clinician factors, and client factors in two of the three cases in which the summary scores indicate insufficient MI quality (Table 10, Cases 11 and 15). And in Cases 8, 9, and 19, in spite of the sufficient MI quality, we did not observe a mechanism of change. In this sample of 24 cases (109 MI sessions), we did not see clear patterns linking the application of clinician factors and the appearance of client factors and of clues for the mechanisms of change to the summary scores of the coaches.

## Discussion

In this study, we explored the components of effective MI for smoking cessation in patients with CAD. We systematically searched for active ingredients (i.e., clinician factors and client factors and their interactions) and subsequent clues for mechanisms of change. In all the sessions, the coaches used eight out of nine clinician factors. None of the coaches employed the ninth clinician factor “creating a change plan,” while this factor is intended to target the self-efficacy of the patient. Notably, the (preparations for the) actual smoking cessation, and the coping with difficult smoking situations afterward, often were conversational topics in anticipation of these situations. These discussions, however, were restricted to general activity and coping strategies, and they never reached the status of a concrete plan with goals, actions, resources, and concrete coping strategies for potential barriers to remain non-smoking.

“Proportion change talk” was the highest prevalent client factor, which was mostly elicited through reflections and open questions directed at smoking cessation behavior or intentions. We observed clues for three out of the four different mechanisms of change, where the mechanism of “changing self-perception” was not observed in this sample. It seems plausible, however, that changing self-perceptions from being “a smoker” to considering oneself as “a non-smoker” is also a mechanism of change for smoking cessation in patients with CAD [see also Miller and Rollnick, 2013, p. 295]. We observed that the majority of the patients felt ambivalent about their ability to stop smoking, and some patients were also ambivalent about their willingness to stop. This means that a MI strategy directed at the mechanisms of change “increasing self-efficacy” and “arguing oneself into change” seems a good fit for MI coaching for smoking cessation in patients with CAD.

Though the clinician factors almost always activated one or more client factors, the interactions between these factors that turn them into active ingredients require a more extensive and more comprehensive strategy, and is more complex, than the mere application of a clinician factor followed by a client factor. For instance, the conversation in Box 3 is a good example. There are many clinician factors (trusting relationship/empathy, eliciting change talk, influencing patient sense making, and supporting autonomy) and many client factors (experiencing a safe environment/opening up, proportion change talk, changing sense making, experiencing autonomy, and resolving ambivalence) involved in that conversation. All these factors interacted and became an active ingredient, which activated the mechanism of change “arguing oneself into change.” This means that the activation of a mechanism of change depends on the tailoring of the MI strategy of the coaches to the individual patient process. Many clinician factors and client factors are involved and interact during a larger part of the session or consecutive sessions, and at some point, they become an active ingredient. In other cases, the same clinician and client factors did not become an active ingredient, probably due to differences in (among other things) the patient process, the timing, the sequence, and/or the exact content of the factor.

Our findings on the mechanisms of change are in accordance with the results of the mediation analysis by Magill et al. (2017), who tested a model with active ingredients, mechanisms of change, and patient outcomes in a population of young heavy alcohol users. They found “increasing motivation to change” and “increasing self-efficacy” as MI-specific mechanisms of change. The mechanisms of change “arguing oneself into change” and “changing self-perceptions” were not tested in this study (Magill et al., 2017). In our previous qualitative study on active ingredients and mechanisms of change in motivational interviewing for medication adherence in a population of patients with schizophrenia (Dobber et al., 2020), we found many clues for the mechanisms of change “arguing oneself into change,” a few for “increasing motivation to change” and one clue for “changing self-perception,” but no clues for “increasing self-efficacy/confidence.” It is plausible that self-efficacy plays a more important role in decreasing alcohol use and in smoking cessation than in medication adherence.

One of the goals for this study was to explore whether MI quality depends on the use of active ingredients by the coaches. The summary scores of the MISC and the SCOPE (see the last five columns of Table 10) are considered to be an indication of the quality of the MI delivered (Martin et al., 2005; Miller et al., 2008; Miller and Rollnick, 2014). In our study, in three cases, the MI quality was insufficient (see Table 10). However, in two of these cases, we did observe mechanisms of change. And, only in nine cases, all scores are at least on the proficiency level, while in three of these cases, we did not observe clues for mechanisms of change. This is explained by the focus of the summary scores, which, except for the global ratings, are mainly targeting the number and proportion of MI techniques executed and not the content of the MI processes: when and how the techniques were executed and their effects on the patient. So, we might consider the presence of an active ingredient in MI as a prerequisite for good MI. If we do, the summary scores fall short and are not enough distinctive. However, due to the definition of “active ingredients” by Nock (2007), we only call (combinations of) clinician factors and client factors “active ingredients” if and when it activates a mechanism of change. In this study, we found that the active ingredients comprised a complex interaction, and, depending on the patient (and maybe also on the coach), different combinations of clinician and client factors constituted the active ingredients. Therefore, in MI, there seem to be no fixed active ingredients, but the active ingredients seem more fluid and personalized. So, though it is difficult to determine the exact characteristics of “good MI,” the presence of active ingredients may be a candidate characteristic.

In our study, there appears to be an association between the patient outcome (smoking status at 12 months) and the presence of observed clues of the mechanisms of change. However, the sample size of this qualitative study is very small, and the study was not designed to detect such a relationship. Therefore, we regard this finding as a stimulus for further investigation only.

### Strengths and Limitations of This Study

This study attempts to open the “black box” of MI counseling for smoking cessation in patients with CAD. We obtained rich qualitative data on both patient and coach processes to strengthen motivation and commitment for smoking cessation, and we identified two important mechanisms of change in MI for smoking cessation in patients with CAD. This study also addresses an important problem for complex behavioral interventions, namely uncertainty of the exact content of the intervention delivered. Using both quantitative and qualitative research methods, we analyzed in detail both the content and the process of the MI sessions. This made it possible to study the complex interaction between clinician and client factors and the activation of the mechanisms of change. This kind of knowledge is important to understand and enhance the application of MI.

Further, our findings add to the debate on what characterizes “good MI.” We identified some shortcomings of the summary scores as a stand-alone criterion for “good MI,” and argue that the presence of active ingredients should be considered as an appropriate addition to the current criteria.

An important limitation is that 27.8% of the sessions were not audiotaped; thus they were not available for analysis. The reasons for not audiotaping these sessions were not recorded. There was no documented refusal of permission to record the session. The Luchtsignaal coaches function in a community-based lifestyle program. In their daily practice, they coach many people on smoking cessation, among whom were the trial patients. Recording these sessions is not part of their routine, and some coaches mentioned that they forgot to record some of the sessions. A likely explanation for the unrecorded sessions is, therefore, that the coaches forgot to record the sessions. This limitation forces us to be prudent in interpreting the data of the incomplete cases, especially concerning the presence or absence of active ingredients and mechanisms of change in some of these cases, the transition probabilities, and the overall MI quality delivered. In addition, the analysis of the transition probabilities using GSEQ 5.1 combines all patient statements in one pool, and all coach statements in another pool, while, in fact, these statements are clustered in 24 coach–patient relations. Furthermore, in this analysis, we calculated only the probability of patient change talk and sustain talk *immediately* following the statements of the coach (lag = 1). However, it is likely that the effects of many statements of the coach will last longer.

Another limitation is that we did not study the relation between active ingredients, mechanism of change, and the actual smoking status at 12 months. This means that we only took a small step by showing the actual presence of active ingredients and mechanisms of change. For causality, many other steps have to be taken (Hill, 1965; Kazdin and Nock, 2003; Nock, 2007): the right temporal relation, experiment, statistical mediation, strong association, specificity, gradient-dose relationship, consistency, plausibility, and coherence.

## Conclusion

Most active ingredients were observed when the coach adapted his/her MI strategy to the individual patient process of change. This created the possibility of several clinician and client factors to interact in such a way that they form an active ingredient and activate a mechanism of change. The combination of targeting the mechanisms of change “increasing self-efficacy” and “arguing oneself into change” seems a good MI strategy in coaching patients with CAD for smoking cessation. This helps patients to solve their ambivalence about their ability to quit smoking and also strengthen the willingness of the patient to quit.

There is more to good quality MI than a trusting relationship and the application of MI-consistent conversational techniques. Although these certainly are prerequisites for effective MI, in quality assessments, the presence or absence of active ingredients should also be taken into account.

## Data Availability Statement

Parts of the dataset generated and analyzed for this study can be found in the Figshare repository: https://doi.org/10.21943/auas.10265054.v1. Parts of the datasets generated and analyzed during the current study are not publicly available due to identifying patient information. Data may be available from the corresponding author upon request, but restrictions apply on the availability of these data in accordance with the ethical rules of the Medical Ethical Committee of the Amsterdam UMC.

## Ethics Statement

The studies involving human participants were reviewed and approved by the Medical Ethics Committee of the Amsterdam UMC, Amsterdam. The patients/participants provided their written informed consent to participate in this study.

## Author Contributions

JD, MS, CL, RP, GR, WS, LH, and BM contributed to the study design and participated in writing the manuscript. JD and MS performed the data acquisition. JD, MS, and GR performed the data analysis. JD, GR, RP, and WS interpreted the data. MS, CL, and BM checked the data interpretation. All authors approved the final manuscript.

## Conflict of Interest

The authors declare that the research was conducted in the absence of any commercial or financial relationships that could be construed as a potential conflict of interest.
