# Supplementary material for: Active Ingredients and Mechanisms of Change in Motivational Interviewing for Smoking Cessation in Patients With Coronary Artery Disease: A Mixed Methods Study
Source: Front Psychol. 2021 Jun 22;12:599203. doi: 10.3389/fpsyg.2021.599203 (PMC8258345; doi:10.3389/fpsyg.2021.599203)
Supplement: Supplementary file 1 [file Data_Sheet_1.docx]

**Supplementary material 1. Clinician factors** (“what the clinician does in the treatment, including clinician behaviors, characteristics, and directives” Nock, 2007, p.8s [1]).

Eliciting change talk [e.g. 2,3]

The coach purposefully employs activities to elicit change talk (e.g. through complex reflections or open ended questions). Change talk entails patient statements in favor of behavior change in the direction of the target behavior: smoking cessation.

Discussing ambivalence and/or barriers [e.g. 4,5]

The coach openly/explicitly or implicitly talks about the patient’s ambivalence towards smoking cessation. The coach may discuss both sides of the ambivalence, or how the ambivalence may be solved, or differentially simply reflect the patient’s concerns on the contra side and reflect and elaborate on the pro-side of change.

The discussion of barriers may concentrate on identifying barriers and on discussing the patient’s concerns about these barriers.

Creating discrepancy / relating values [e.g. 2,6]

The coach tries to direct the course of the conversation in such a way that the patient relates his/her own values or life goals to the target behavior (smoking cessation). In doing so, the coach may attempt to create discrepancy, i.e. the patient experiences a gap between the present situation and the desired situation.

Building a trusting relationship / empathy [e.g. 7,8]

Coach and patient develop a relationship of mutual respect and trust. The coach takes a listening and empathic stance, shows genuine interest in the patient, and sympathizes with the patient’s experiences (and does not pursue his/her own agenda). The patient is being listened to, understood, or the coach takes an effort to understand him/her.

Influencing patient’s sense making [e.g. 9,10]

By providing information, the coach attempts to influence the patient’s sense making (in which the present behavior [smoking] seems obvious and logical to the patient) in such a way that the patient finds his/her sense making no longer logical, or even incorrect.

Supporting self-efficacy / competency [e.g. 2,11]

The coach promotes or affirms the patient’s experience of competency or confidence or the belief of self-efficacy, e.g. by discussing coping strategies to handle these barriers.

Supporting autonomy [e.g. 2,7]

The coach promotes or affirms that the patient is the only person who decides (about smoking cessation); or promotes or affirms that the patient is gaining control or has control over the smoking; or promotes or affirms the patient’s (feeling of) autonomy.

Creating a change plan [e.g. 12,13]

The coach and the patient work out a concrete plan that fits the patient’s actions and strategies for smoking cessation or to avoid or cope with potential barriers to persist in non-smoking.

Supporting self-esteem [e.g. 2,14]

The coach emphasizes (e.g. affirms or reflects) a positive patient trait or skill.

**References**

1. Nock MK. Conceptual and design essentials for evaluating mechanisms of change. Alcohol Clin Exp Res. 2007;31(S3):4S-12S. doi: 10.1111/j.1530-0277.2007.00488.x
2. Miller WR, Rollnick S. Motivational interviewing: helping people change. 3^rd^ ed. New York: Guilford Press (2013).
3. Glynn LH, Moyers TB. Chasing change talk: the clinician’s role in evoking client language about change. J Subst Abuse Treat (2010) 39:65-70. doi: 10.1016/j.jsat.2010.03.012
4. Magill M, Kiluk BD, McCrady B, Tonigan JS, Longabaugh R. Active ingredients of treatment and client mechanisms of change in behavioural treatments for alcohol use disorders: progress 10 years later. Alcohol Clin Exp Res (2015) 39;1852-62. doi: 10.1111/acer.12848
5. Miller WR, Rollnick S. Talking oneself into change: motivational interviewing, stages of change, and therapeutic process. J Cogn Psychother (2004) 18:299-308. doi: 10.1891/jcop.18.4.299.64003
6. McNally AM, Palfai TP, Kahler CW. Motivational interventions for heavy drinking college students: examining the role of discrepancy-related psychological processes. Psychol Addict Behav (2005) 19:79-87. doi: 10.1037/0893-164X.19.1.79
7. Copeland L, McNamara R, Kelson M, Simpson S. Mechanisms of change within motivational interviewing in relation to health behaviors outcomes: a systematic review. Patient Educ Couns (2015) 98;401-11. doi: 10.1016/j.pec.2014.11.022
8. Moyers TB, Miller WR. Is low therapist empathy toxic? Psychol Addict Behav (2013) 27:878-84. doi: 10.1037/a0030274
9. Berger BA, Villaume WA. Motivational interviewing for health care professionals. A sensible approach. Washington DC: American Pharmacists Association (2013).
10. Berger BA, Bertram CT. Motivational interviewing and specialty pharmacy. J Manag Care Spec Pharm (2015) 21:13-7. doi: 10.18553/jmpc.21.1.13
11. Barnett NP, Apodaca TR, Magill M, Colby SM, Gwaltney C, Rohsenow DJ, et al. Moderators and mediators of two brief interventions for alcohol in the emergency department. Addiction (2010) 105:452-65. doi: 10. 1111/j.1360-0443.2009.02814.x
12. Gollwitzer PM. Implementation intentions. Strong effects of simple plans. Am Psychol (1999) 54:493-503. doi: 10.1037/0003-066X.54.7.493
13. Lee CS, Baird J, Longabaugh R, Nirenberg TD, Mello MJ, Woolard R. Change plan as an active ingredient of brief motivational interviewing for reducing negative consequences of drinking in hazardous drinking emergency-department patients. J Stud Alcohol Drugs (2010) 71:726-33. doi: 10.15288/jsad.2010.71.726
14. Miller WR. Motivational interviewing with problem drinkers. Behav Psychother (1983) 11:147-72. doi: 10.1017/S0141347300006583
