# Supplementary material for: Active Ingredients and Mechanisms of Change in Motivational Interviewing for Smoking Cessation in Patients With Coronary Artery Disease: A Mixed Methods Study
Source: Front Psychol. 2021 Jun 22;12:599203. doi: 10.3389/fpsyg.2021.599203 (PMC8258345; doi:10.3389/fpsyg.2021.599203)
Supplement: Supplementary file 2 [file Data_Sheet_2.docx]

**Supplementary material 2. Client factors** (“what the client does in treatment, including behaviors, characteristics, and verbalizations on their part” (Nock, 2007, p.8s [1]).

Change talk [e.g. 2,3]

Patient statements in favor of change: smoking cessation. Patient statements may concern desire, ability, reasons, need, commitment, taking steps, and other pro-change statements.

Resolving ambivalence [e.g. 4,5]

The patient expresses a choice for one of the two sides of ambivalence, which resolves the originally present doubt or ambivalence.

Changing sense making [e.g. 6,7]

The patient adjusts his/her original explanatory model through which the patient explains his/her medical and psychological condition and his/her coping with it, including his/her sense making of smoking.

Or: the patient adjusts his/her reasoning about the consequences of the explanatory model for his/her coping with this condition, including his/her sense making of smoking.

Experiencing autonomy [e.g. 4,8]

The patient’s reaction shows that, due to a statement made by the coach, the patient experiences autonomy or being in control, in an enhanced degree.

Experiencing discrepancy [e.g. 9,10]

Patient’s statements show that the patient experiences a gap (or the development of a gap) between the existent situation and the desired situation, recognizing certain life goals or values for which smoking cessation can be key to accomplish (a higher degree of) these life goals or values.

Experiencing a safe environment / opening up [e.g. 4,11]

The patient overtly talks about his/her concerns and opens up. The patient goes deeper into personal material, spontaneously elaborating on it with feeling.

In-depth self-exploration [e.g. 8,12]

The patient explores personally relevant material and may discover new perspectives and/or personal meanings (see also: Client self-exploration, MISC2.1, p.7; 2008 [13]).

Experiencing competency / self-efficacy [e.g. 4,14]

Patient statement show that the patient experiences an enhanced degree of competence or self-efficacy in his/her ability to quit smoking.

Readiness to change [e.g. 4,15]

The patient states directly or indirectly to quit smoking (while up to that moment, he/she was not willing and/or able to quit smoking).

**References**

1. Nock MK. Conceptual and design essentials for evaluating mechanisms of change. Alcohol Clin Exp Res. 2007;31(S3):4S-12S. doi: 10.1111/j.1530-0277.2007.00488.x
2. Copeland L, McNamara R, Kelson M, Simpson S. Mechanisms of change within motivational interviewing in relation to health behaviors outcomes: a systematic review. Patient Educ Couns (2015) 98;401-11. doi: 10.1016/j.pec.2014.11.022
3. Magill M, Apodaca TR, Borsari B, Gaume J, Hoadley A, Gordon REF, et al. A meta-analysis of motivational interviewing process: technical, relational, and conditional process models of change. J Consult Clin Psychol (2018) 86:140-57. doi: 10.1111/acer.12848
4. Miller WR, Rollnick S. Motivational interviewing: helping people change. 3^rd^ ed. New York: Guilford Press (2013).
5. Miller WR, Rollnick S. Talking oneself into change: motivational interviewing, stages of change, and therapeutic process. J Cogn Psychother (2004) 18:299-308. doi: 10.1891/jcop.18.4.299.64003
6. Berger BA, Villaume WA. Motivational interviewing for health care professionals. A sensible approach. Washington DC: American Pharmacists Association (2013).
7. Berger BA, Bertram CT. Motivational interviewing and specialty pharmacy. J Manag Care Spec Pharm (2015) 21:13-7. doi: 10.18553/jmpc.21.1.13
8. Miller WR. Motivational interviewing with problem drinkers. Behav Psychother (1983) 11:147-72. doi: 10.1017/S0141347300006583
9. Apodaca TR, Longabaugh R. Mechanisms of change in motivational interviewing: a review and preliminary evaluation of the evidence. Addiction (2009) 104:705-15. doi: 10.1111/j.1360-0443.2009.02527.x
10. McNally AM, Palfai TP, Kahler CW. Motivational interventions for heavy drinking college students: examining the role of discrepancy-related psychological processes. Psychol Addict Behav (2005) 19:79-87. doi: 10.1037/0893-164X.19.1.79
11. Arkowitz H, Miller WR, Westra HA, Rollnick S. Motivational interviewing in the treatment of psychological problems. New York: The Guilford Press (2008). p.324-42.
12. Apodaca TR, Borsari B, Jackson KM, Magill M, Longabaugh R, Mastroleo NR, et al. Sustain talk predicts poorer outcomes among mandated college student drinkers receiving a brief motivational intervention. Psychol Addict Behav (2014) 28:631-38. doi: 10.1037/a0037296
13. Miller WR, Moyers TB, Ernst D, Amrhein P. Manual for the Motivational Interviewing Skill Code (MISC). Version 2.1. 2008. <https://casaa.unm.edu/download/misc.pdf> Accessed 27 October 2013.
14. Gaume J, Gmel G, Daeppen JB. Brief alcohol interventions: do counsellors’ and patients’ communication characteristics predict change? Alcohol Alcohol (2008) 43:62-9. doi: 10.1093/alcalc/agm141
15. Miller WR, Rose GS. Toward a theory of motivational interviewing. Am Psychol (2009) 64:527-37. doi: 10.1037/a0016830
