# Supplementary material for: Active Ingredients and Mechanisms of Change in Motivational Interviewing for Smoking Cessation in Patients With Coronary Artery Disease: A Mixed Methods Study
Source: Front Psychol. 2021 Jun 22;12:599203. doi: 10.3389/fpsyg.2021.599203 (PMC8258345; doi:10.3389/fpsyg.2021.599203)
Supplement: Supplementary file 3 [file Data_Sheet_3.docx]

**Supplementary material 3. Hypothetical mechanisms of change** (“the processes that emerge from or occur as a result of the clinician and client factors, and their interaction, that explain how those factors lead to change in the outcomes of interest” Nock, 2007, p.8s [1]).

Arguing oneself into change [e.g. 2,3]

The patient talks about smoking cessation in such a way that he/she convinces him/herself to quit smoking (while up to that moment he/she was unconvinced). If the patient was already convinced and/or had already stopped smoking, he/she may strengthen the belief to remain non-smoking.

Increasing motivation to change [e.g. 2,4]

The patient clearly expresses a stronger motivation for smoking cessation than earlier in the same session or in previous sessions.

Increasing self-efficacy / confidence [e.g. 2,5]

The patient expresses an enhanced degree of self-efficacy or confidence in his/her ability to remain non-smoking.

Changing self-perception [e.g. 6,7]

The patient’s statements show a shift in self-perception regarding (an aspect related to) smoking.

**References**

1. Nock MK. Conceptual and design essentials for evaluating mechanisms of change. Alcohol Clin Exp Res. 2007;31(S3):4S-12S. doi: 10.1111/j.1530-0277.2007.00488.x
2. Miller WR, Rollnick S. Motivational interviewing: helping people change. 3^rd^ ed. New York: Guilford Press (2013).
3. Miller WR, Rollnick S. Talking oneself into change: motivational interviewing, stages of change, and therapeutic process. J Cogn Psychother (2004) 18:299-308. doi: 10.1891/jcop.18.4.299.64003
4. Copeland L, McNamara R, Kelson M, Simpson S. Mechanisms of change within motivational interviewing in relation to health behaviors outcomes: a systematic review. Patient Educ Couns (2015) 98;401-11. doi: 10.1016/j.pec.2014.11.022
5. Moos RH. Theory-based active ingredients of effective treatments for substance use disorders. Drug Alcohol Depend (2007) 88:109-21. doi: 10.1016/drugalcdep.2006.10.010
6. Miller WR, Rollnick S. Motivational interviewing: preparing people for change. 2^nd^ ed. New York: Guilford Press (2002).
7. Bem DJ. Self-perception: an alternative interpretation of cognitive dissonance phenomena. Psychiatry Rev (1967) 74:183-200. doi: 10.1037/h0024835
